# Ascophyllan Induces Activation of Natural Killer Cells in Mice In Vivo and In Vitro

**DOI:** 10.3390/md17040197

**Published:** 2019-03-28

**Authors:** Wei Zhang, Takasi Okimura, Tatsuya Oda, Jun-O Jin

**Affiliations:** 1 Scientific Research Center, Shanghai Public Health Clinical Center & Institutes of Biomedical Sciences, Shanghai Medical College, Fudan University, Shanghai 201508, China; weiwei061215@126.com; 2 Research and Development Division, Hayashikane Sangyo Co., Ltd., Shimonoseki, Yamaguchi 750-8608, Japan; tokimura@hayashikane.co.jp; 3 Graduate School of Fisheries Science and Environmental Studies, Nagasaki University, Nagasaki 852-8521, Japan; t-oda@nagasaki-u.ac.jp; 4 Department of Medical Biotechnology, Yeungnam University, Gyeongsan 38541, Korea

**Keywords:** ascophyllan, fucoidan, natural killer cells, IFN-γ, cytotoxicity

## Abstract

Natural marine polysaccharides have demonstrated immune stimulatory effects in both mice and humans. Our previous study compared the ability of ascophyllan and fucoidan to activate human and mouse dendritic cells (DCs). In this study, we further examined the effect of ascophyllan on the activation of mouse natural killer (NK) cells in vivo and in vitro and compared it to that of fucoidan, a well-studied natural marine polysaccharide. Specifically, administration of ascophyllan to C57BL/6 mice increased the number of NK cells in the spleen when compared to the number in PBS-treated mice. Moreover, the number of IFN-γ-producing NK cells and expression of CD69 were markedly upregulated by ascophyllan treatment. Ascophyllan treatment also induced IFN-γ production and CD69 upregulation in isolated NK cells, but did not promote cell proliferation. Finally, ascophyllan treatment increased the cytotoxicity of NK cells against Yac-1 cells. The effects of ascophyllan on NK cell activation were considerably stronger than those of fucoidan. These data demonstrated that ascophyllan promotes NK cell activation both in mice and *in vitro*, and its stimulatory effect on NK cells is stronger than that of fucoidan.

## 1. Introduction

Natural marine polysaccharides show various bioactivities, including anti-diabetic, anti-inflammatory, anti-bacterial, anti-viral, and immunostimulatory activities [1,2]. Moreover, plant metabolites are well conserved from algae to higher plants, indicating that these molecules are of great biological value to these organisms [3,4]. Polysaccharide components from algae have immunomodulatory effects in humans and mice. In particular, fucoidan, a well-studied marine polysaccharide, can activate dendritic cells (DCs), natural killer (NK) cells, neutrophils, and T cells [5,6,7,8]. Recently, we showed that ascophyllan purified from *Ascophyllum nodosum* (*A. nodosum*) had immune cell activation effects that were comparable to those of fucoidan [9,10,11]. Ascophyllan is a heterogeneous sulfated polysaccharide that is clearly distinguishable by its monosaccharide composition from fucoidan, which mainly contains fucose as its sugar component [12,13]. Ascophyllan has been shown to activate mouse bone marrow-derived DCs (BMDCs) and splenic DCs, and its effects are stronger than those of fucoidan [11]. Moreover, pro-inflammatory cytokine levels in mouse serum are much higher following ascophyllan treatment than those following fucoidan treatment [11]. Although the previous study examined the effects of ascophyllan on DC activation, the stimulatory effect of ascophyllan on NK cells has not been studied or compared to that of fucoidan.

NK cells are innate immune cells that play protective roles against bacterial and viral infection [14,15]. Infected or damaged cells are recognized by pathogen-associated molecular patterns or damage-associated molecular patterns, respectively, and are presented on their surface to promote the activation of NK cells [16,17]. NK cells are the primary producers of interferon-γ (IFN-γ), which is secreted following stimulation of the corresponding surface receptors [18,19]. IFN-γ expression by NK cells contributes to the activation of other immune cells and subsequent pathogen elimination [18]. Moreover, regulation of target cell elimination by NK cells depends on the expression levels of major histocompatibility complex (MHC) class I molecules [20,21,22]. Virus-infected cells and cancer cells express low levels of MHC class I molecules and can be targeted for elimination by NK cells [20]. Activation of NK cells is mediated not only by pathogens and damaged cells but also by other immune cells, such as DCs and macrophages [23,24,25]. Type I IFNs, which are produced by DCs, macrophages, and other NK cells, function to activate NK cell functions, including cytotoxicity and IFN-γ production [26,27].

Although it was previously shown that the ability of ascophyllan to induce DC activation was much stronger than that of fucoidan [11], the effects of ascophyllan on NK cell activation is yet to be studied. In this study, we evaluated the effect of ascophyllan on NK cell activation in mice and compared it with the effects of fucoidan.

## 2. Results and Discussion

### 2.1. Ascophyllan Promotes the Proliferation of NK Cells in Mice

**Figure 1:**
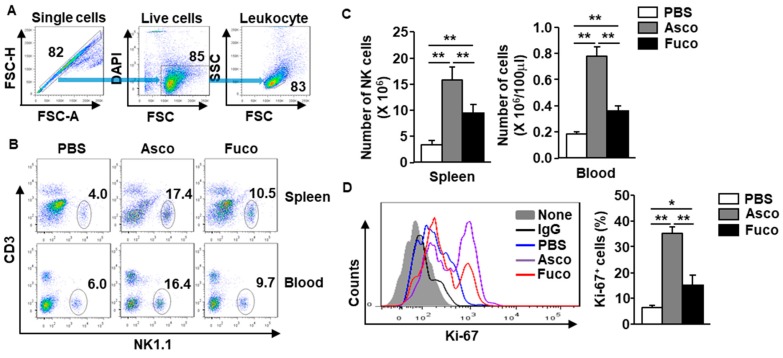
Proliferation of natural killer (NK) cells is upregulated by ascophyllan in mice. Either ascophyllan or fucoidan (50 mg/kg, each) was intraperitoneally (*i.p.*) administered to C57BL/6 mice, and NK cells were analyzed 6 h after administration. (**A**) The gating strategy for flow cytometry is shown. (**B**) Frequencies/percentages of NK1.1^+^CD3^−^ cells in the spleen (upper panel) and blood (lower panel) are shown. (**C**) Absolute numbers of NK1.1^+^CD3^−^ cells in the spleen (left panel) and blood (right panel) are shown. (**D**) Expression of Ki-67 on spleen NK cells (left panel). The mean percentage of Ki-67^+^ cells is shown (right panel). Data represent the average of six samples (two mice in each group from three independent experiments). * *p* < 0.05, ** *p* < 0.01.

To evaluate the stimulatory effect of ascophyllan on NK cell proliferation, C57BL/6 mice were intraperitoneally (*i.p.*) administered either ascophyllan or fucoidan (50 mg/kg, each). NK cells were defined as NK1.1^+^CD3^−^ cells among the leukocytes (Figure 1A, B). Treatment with ascophyllan increased the frequency of NK1.1^+^CD3^−^ cells in the spleen and blood compared to those in mice treated with PBS at 6 h after injection (Figure 1B). In addition, the numbers of NK cells in the spleen and blood were also significantly increased following ascophyllan treatment (Figure 1C). Proliferating NK cells were identified by their surface expression of Ki-67, a marker of proliferating cells, detected at 6 h after ascophyllan or fucoidan treatment. It was found that ascophyllan treatment efficiently increased the number of Ki-67-positive cells compared to the number in the PBS-treated control mice (Figure 1D).

Since the effect of fucoidan on NK cell activation has been well studied [5], we compared the proliferation-inducing abilities of ascophyllan and fucoidan. As shown in Figure 1, ascophyllan treatment had a much greater proliferation-inducing effect in NK cells than fucoidan. These data indicate that ascophyllan can induce NK cell proliferation and the effect is much stronger than that of fucoidan.

### 2.2. Ascophyllan Activates NK Cells in Mice

**Figure 2:**
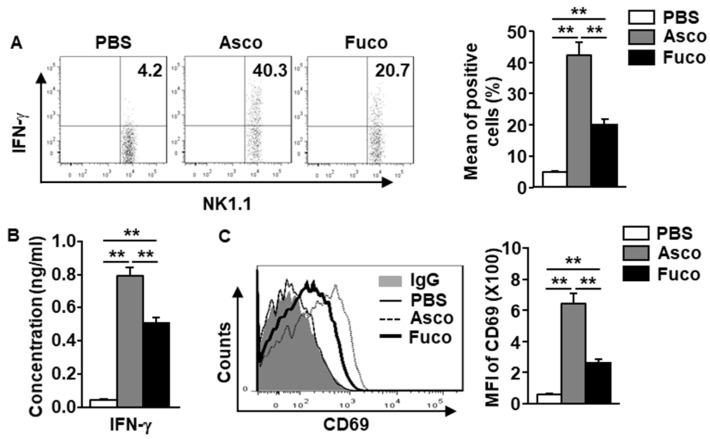
Ascophyllan activates NK cells in mice. Mice were injected with either ascophyllan (Asco, 50 mg/kg) or fucoidan (Fuco, 50 mg/kg). Six hours after injection, the spleens were harvested, and the splenocytes were incubated in a monensin solution for 4 h. (**A**) Intracellular IFN-γ levels in spleen NK cells (left panel). Mean percentage of IFN-γ-producing NK cells (right panel). (**B**) Serum concentration of IFN-γ 6 h after either ascophyllan or fucoidan treatment. (**C**) CD69 expression levels in spleen NK cells (left panel) 6 h after treatment. Mean fluorescence intensity (MFI) of CD69 levels (right panel). Data represent the mean ± standard error of the mean (SEM) of six samples from three independent experiments, ** p < 0.01.

Our finding that ascophyllan promotes NK cell proliferation prompted us to examine the effect of ascophyllan on activating NK cells. Either ascophyllan or fucoidan (50 mg/kg, each) was administered *i.p.* to C57BL/6 mice. Six hours after administration, the spleens were harvested, and the splenocytes were incubated in a monensin solution for an additional 4 h. The results showed that ascophyllan treatment upregulated the intracellular production of IFN-γ in spleen NK cells (Figure 2A). In addition, the serum concentration of IFN-γ was dramatically increased by ascophyllan treatment compared to that induced by PBS (Figure 2B). Further, the expression of the surface marker CD69 on active NK cells was substantially upregulated by ascophyllan (Figure 2C). Consistent with its proliferation-inducing effects, ascophyllan also induced IFN-γ production and CD69 expression in NK cells more strongly than fucoidan. These data suggest that ascophyllan activates spleen NK cells, and its effects are stronger than those of fucoidan.

Fucoidan isolated from *Fucus vesiculosus* (*F. vesiculosus*) was studied in humans and mice for its immunomodulatory effects [8,28]. Although fucoidan from *F. vesiculosus* showed immunostimulatory effects on DC and NK cells, the effects of fucoidan from *Macrocystis pyrifera* (*M. pyrifera*) on DC and NK cell activation were stronger [5]. In this study, we found that mouse NK cell activation by ascophyllan was stronger than that by fucoidan from *M. pyrifera*. Based on a composition study, fucoidan from *M. pyrifera* contained much higher uronic acid (UA) content than fucoidan from *F. vesiculosus* [5]. Interestingly, ascophyllan also contained higher levels of UA than other fucoidans [11,13]. Therefore, the UA content may contribute to its NK cell-activation effects. We will examine the effects of UA on the activation of NK cells and DCs in a future study.

### 2.3. Ascophyllan Directly and Indirectly Activates NK Cells

**Figure 3:**
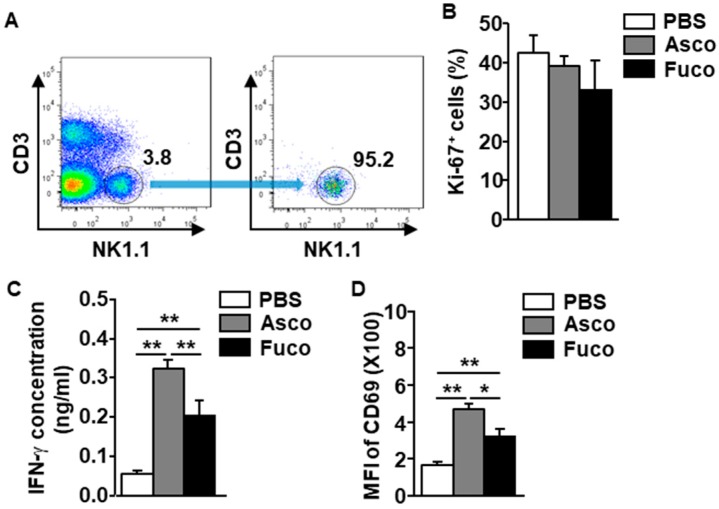
Ascophyllan activates isolated NK cells. NK cells were isolated from C57BL/6 mice, and the cells were incubated with either ascophyllan (Asco, 50 μg/mL) or 50 μg/mL fucoidan (Fuco, 50 μg/mL). (**A**) Percentages of NK1.1^+^CD3^−^ cells in the splenocytes (left panel) and the purity of the NK cells (right panel) are shown. (**B**) Ki-67 expression levels in NK cells were measured 6 h after treatment. (**C**) IFN-γ concentrations in culture medium are shown. (**D**) Surface expression of CD69 was measured in NK cells. Mean ± standard error of the mean (*n* = 6). * *p* < 0.05. ** *p* < 0.01.

In the mouse, many immune cell types are targeted by stimuli, including DCs, macrophages, NK cells, and T cells [29,30,31]. These stimulated immune cells contribute to the activation of other immune cells through cytokine production and cell-to-cell interactions [29,30]. Therefore, we next evaluated the ability of ascophyllan to activate NK cells in mice either directly or indirectly through other stimulated immune cells. As shown in Figure 3A, to evaluate the direct effect of ascophyllan on NK cell activation, NK1.1^+^CD3^−^ NK cells were isolated from the leukocytes in the spleen of naïve mice and treated with either ascophyllan or fucoidan (50 μg/mL, each). The Ki-67 staining levels on the isolated NK cells were not increased by either ascophyllan or fucoidan (Figure 3B). However, the levels of IFN-γ secreted into the culture medium of NK cells were dramatically increased by ascophyllan (Figure 3C). Further, CD69 expression in isolated NK cells was also upregulated by ascophyllan (Figure 3D). Consistent with the in vivo mouse study results, ascophyllan treatment also induced much higher levels of IFN-γ production and CD69 expression than fucoidan. These data indicate that ascophyllan activates NK cells directly but cannot promote the proliferation of NK cells without the aid of other immune cells.

DCs and macrophages have been shown to contribute to NK cell activation in humans and mice [32,33]. Antigen stimulation of DCs and macrophages promotes the production of pro-inflammatory cytokines, such as IL-1, IL-12, IL-15, IL-18, TNF-α, and IFNs [34]. IL-15 promotes the survival and differentiation of NK cells [25,35,36], and is mainly produced by activated DCs [37,38]. Moreover, IL-12 secreted by mature DCs enhances the cytotoxic activity and IFN-γ production of NK cells [39]. In addition, IL-2 produced by activated CD4^+^ T cells and DCs is a potential inducer of NK cell proliferation [23,25]. Although cytokines contribute to the activation and proliferation of NK cells, mature DCs directly interact with NK cells to induce the activation and survival of the latter [40]. In previous studies, ascophyllan activated spleen- and lymph node (LN)-derived DCs and T cells in mice [10]. DCs activated by ascophyllan produced high amounts of pro-inflammatory cytokines, including IL-1, IL-12, and TNF-α [10,11]. In this study, we found that ascophyllan promoted NK cell activation and proliferation in mice. However, the proliferation-promoting effect of ascophyllan on isolated NK cells was diminished; therefore, the effect of ascophyllan on the induction of NK cell proliferation may be mediated by activated DCs and cytokines.

### 2.4. Ascophyllan Enhances the Cytotoxic Activity of NK Cells

**Figure 4:**
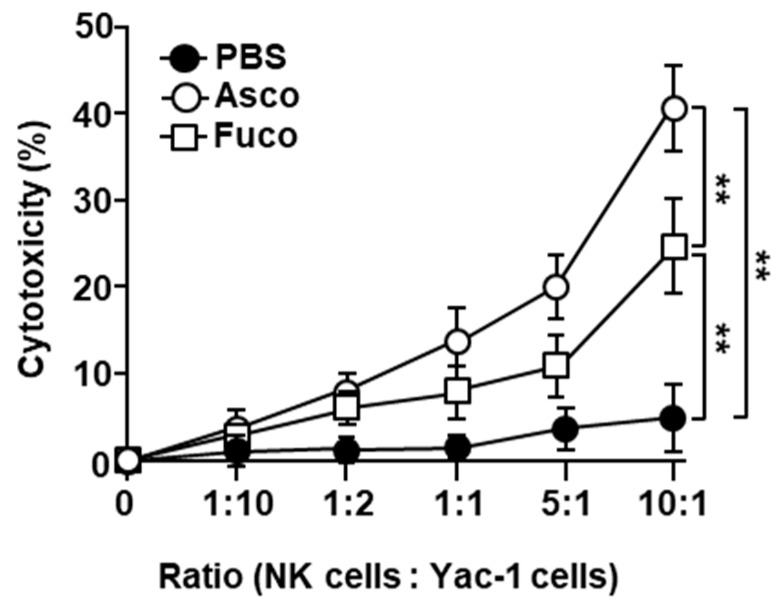
Ascophyllan enhances the cytotoxic activity of NK cells. C57BL/6 mice were administered either ascophyllan (Asco, 50 mg/kg) or fucoidan (Fuco, 50 mg/kg) by *i.p.* injection, twice, separated by 24 h. Ascophyllan- or fucoidan-stimulated spleen NK cells (effector cells) were isolated from mice 24 h after the last injection and co-cultured with Yac-1 cells (target cells) at the indicated ratios. Cytotoxicity was measured after 6 h of co-culture by the lactate dehydrogenase (LDH) assay. Mean ± standard error of the mean. (*n* = 6) ** *p* < 0.01.

Next, we examined whether ascophyllan treatment enhanced the cytotoxic activity of NK cells. C57BL/6 mice were *i.p.* administered either ascophyllan or fucoidan (50 mg/kg, each), and then injected with the same concentration of either ascophyllan or fucoidan 24 h later. One day after the last injection, NK cells were isolated from the splenocytes and co-cultured with Yac-1 cells, a mouse lymphoma cell line [41]. The ascophyllan-treated spleen NK cells showed significantly enhanced cytotoxic activity compared to NK cells from untreated mice (Figure 4). The cytotoxic activity of ascophyllan-induced NK cells was stronger than that of fucoidan-treated NK cells (Figure 4). These data demonstrate that ascophyllan enhances NK cell-mediated cytotoxicity against Yac-1 cells.

The most important function of NK cells is their cytotoxic activity against pathogens [42,43], and NK cell-mediated cytotoxicity is dependent on the expression of MHC class I molecules [42,43]. At steady state, cells present high levels of self-antigen on MHC class I receptors, which prevents attack by immune cells. In cancer cells and virus-infected cells, MHC class I levels are markedly lower than the levels in healthy cells, and thus they can be targets of NK cells [44,45]. Although the MHC class I expression levels on these cells were low, NK cells could not effectively kill these cells without being activated [46]. In this study, we found that ascophyllan enhanced the cytotoxic activity of NK cells, which was even stronger than the activity of fucoidan-activated NK cells. Therefore, ascophyllan may be able to protect mice from cancer and viral infection.

## 3. Materials and Methods

### 3.1. Mice

C57BL/6 mice were provided by the Shanghai Public Health Clinical Center (SPHCC). The mice were housed under pathogen-free conditions, and the mouse room was maintained at 20–22 °C and 50%–60% humidity. This study was conducted in strict accordance with the recommendations outlined in the Guide for the Care and Use of Laboratory Animals of the SPHCC. The Committee on the Ethics of Animal Experiments of SPHCC approved this study (Approval number: SYXK-2010-0098).

### 3.2. Antibodies and Reagents

Ascophyllan was purified from *A. nodosum* as a sulfated fucan preparation [13,47]. Briefly, in the first step, a hot water extraction was performed to obtain the total soluble polysaccharide fraction, which contained ascophyllan, fucoidan, and trace levels of alginate. In the second step, alginate was removed by alginate lyase digestion. The third step was the selective precipitation of ascophyllan in the presence of NaCl and alcohol. In this step, ascophyllan was separated from fucoidan. Finally, a composition analysis was performed to characterize the isolated polysaccharides [45]. Fucoidan from *M. pyrifera* was obtained from Sigma Aldrich (St. Louis, MO, USA). Fluorescence-conjugated Abs against IgG1, IgG2a, CD3 (17A2), CD69 (H1.2F3), NK1.1 (PK136), Ki-67 (16A8), and IFN-γ (XMG1.2) were purchased from BioLegend (San Diego, CA, USA).

### 3.3. NK Cell Analysis

The spleen was cut into small fragments with curved scissors and digested in digestion buffer (RPMI 1640 containing 2% fetal bovine serum [FBS] and collagenase IV) for 20 min at room temperature. The digested tissue was washed with 10 ml of PBS and resuspended in 5 ml of histopaque 1077 (Sigma Aldrich). An additional 5 ml of histopaque 1077 was added to the upper layer, and 1 mL of FBS was layered over the histopaque. The suspended cells were then centrifuged at 3000 RPM for 10 min. Splenocytes were obtained from the light density fraction (<1.077 g/cm^3^). The cells were then stained with anti-CD3-FITC and anti-NK1.1-PE antibodies (BioLegend, San Diego, CA, USA), and the NK1.1^+^CD3^−^cells were analyzed by flow cytometry (Becton Dickinson, San Diego, CA, USA).

### 3.4. Ex Vivo Cell Stimulation and Intracellular Cytokine Staining

C57BL/6 mice were administered ascophyllan or fucoidan. Six hours after administration, their spleens were harvested, and splenocytes or isolated NK cells were incubated with monensin (BioLegend, San Diego, CA, USA) for 4 h. After washing with PBS, the cells were stained for surface antibodies, fixed with fixation buffer, and permeabilized with Cytofix/Cytoperm buffer (eBioscience, San Diego, CA, USA). Subsequently, they were stained with anti-IFN-γ antibodies in Perm/Wash buffer (eBioscience, San Diego, CA, USA) for 30 min. In all experiments, an isotype control IgG antibody was used as a negative control.

### 3.5. ELISA

An IFN-γ ELISA kit was purchased from BioLegend (San Diego, CA, USA). IFN-γ concentrations in the serum and culture medium were measured in triplicate using the ELISA kit.

### 3.6. NK Cell Isolation

NK cells were purified from splenocytes using a mouse NK cell isolation kit (Miltenyi Biotec Auburn, CA, USA). The purity of the isolated cells was measured by flow cytometry (FACS Fortessa; Becton Dickinson, Franklin Lakes, NJ, USA) and was found to be greater than 95%.

### 3.7. Cytotoxicity Assay

As previously described [48], NK cells were incubated with Yac-1 cells (ATCC, Manassas, VA, USA), a mouse lymphoma cell line, for 24 h. Cytotoxicity was determined by the lactate dehydrogenase (LDH) assay [49] (Roche, Basel, Switzerland) according to the manufacturer’s instructions.

### 3.8. Statistical Analysis

The results are expressed as the mean ± SEM. One-way ANOVA was performed to analyze the data, with Tukey’s multiple comparison test, using GraphPad Prism 4. *P* values less than 0.05 were considered statistically significant.

## 4. Conclusions

In this study, we demonstrated that ascophyllan could induce the activation and proliferation of mouse NK cells in vitro and in vivo. The ascophyllan-activated NK cells produced IFN-γ and exhibited cytotoxic activity against cancer cells. Therefore, ascophyllan could be used as an immunostimulatory molecule for the treatment of cancer and infectious diseases.
